# No Evidence for an Item Limit in Change Detection

**DOI:** 10.1371/journal.pcbi.1002927

**Published:** 2013-02-28

**Authors:** Shaiyan Keshvari, Ronald van den Berg, Wei Ji Ma

**Affiliations:** Department of Neuroscience, Baylor College of Medicine, Houston, Texas, United States of America; New York University, United States of America

## Abstract

Change detection is a classic paradigm that has been used for decades to argue that working memory can hold no more than a fixed number of items (“item-limit models”). Recent findings force us to consider the alternative view that working memory is limited by the precision in stimulus encoding, with mean precision decreasing with increasing set size (“continuous-resource models”). Most previous studies that used the change detection paradigm have ignored effects of limited encoding precision by using highly discriminable stimuli and only large changes. We conducted two change detection experiments (orientation and color) in which change magnitudes were drawn from a wide range, including small changes. In a rigorous comparison of five models, we found no evidence of an item limit. Instead, human change detection performance was best explained by a continuous-resource model in which encoding precision is variable across items and trials even at a given set size. This model accounts for comparison errors in a principled, probabilistic manner. Our findings sharply challenge the theoretical basis for most neural studies of working memory capacity.

## Author Summary

Working memory is a fundamental aspect of human cognition. It allows us to remember bits of information over short periods of time and make split-second decisions about what to do next. Working memory is often tested using a change detection task: subjects report whether a change occurred between two subsequent visual images that both contain multiple objects (items). The more items are present in the images, the worse they do. The precise origin of this phenomenon is not agreed on. The classic theory asserts that working memory consists of a small number of slots, each of which can store one item; when there are more items than slots, the extra items are discarded. A modern model postulates that working memory is fundamentally limited in the quality rather than the quantity of memories. In a metaphor: instead of watering only a few plants in our garden, we water all of them, but the more plants we have, the less water each will receive on average. We show that this new model does much better in accounting for human change detection responses. This has consequences for the entire field of working memory research.

## Introduction

Visual working memory, the ability to buffer visual information over time intervals of the order of seconds, is a fundamental aspect of cognition. It is essential for detecting changes [1]–[3], integrating information across eye fixations [4]–[5], and planning goal-directed reaching movements [6]. Numerous studies have found that visual working memory is limited, but the precise nature of its limitations is subject of intense debate [7]–[14]. The standard view is that visual working memory cannot hold more than about four items, with any excess items being discarded [7]–[9], [15]–[18]. According to an alternative hypothesis, working memory limitations take the form of a gradual decrease in the encoding precision of stimuli with increasing set size [10]–[11], [13], [19]–[23]. In this view, encoding precision is a continuous quantity, and this hypothesis has therefore also been referred to as the continuous-resource hypothesis.

Historically, the leading paradigm for studying visual working memory has been change detection, a task in which observers report whether a change occurred between two scenes separated in time [2]–[3], [24]. Not only humans, but also non-human primates can perform multiple-item change detection [25]–[28], and physiological studies have begun to investigate the neural mechanisms involved in this task [27]. Findings from change detection studies have been used widely to argue in favor of the item-limit hypothesis [2], [8], [15]–[18]. The majority of these studies, however, used stimuli that differed categorically from each other, such as line drawings of everyday objects or highly distinct and easily named colors. The logic is that for such stimuli, changes are large relative to the noise, avoiding the problem of “comparison errors” [1], [18], [29]–[30] that would be associated with low encoding precision (high noise). When encoding precision is limited, an observer's stimulus measurements are noisy and will differ between displays for each item, even if the item did not change. The observer then has to decide whether a difference in measurements is due to noise only or to a change plus noise, which is especially problematic when changes are small. This signal detection problem results in comparison errors.

Attempts to avoid such errors by using categorical stimuli run into two objections: first, using such stimuli does not guarantee that comparison errors are absent and can be ignored in modeling; second, there is no good reason to avoid comparison errors, since the pattern of such errors can help to distinguish models. Ideally, change detection performance should be measured across a wide range of change magnitudes, including small values, as we do here. Comparison errors can, in fact, be modeled rather easily within the context of a Bayesian-observer model. Bayesian inference is the decision strategy that maximizes an observer's accuracy given noisy measurements [31]–[32], and was recently found to describe human decision-making in change detection well [33].

We conducted two change detection experiments, in the orientation and color domains, in which we varied both set size and the magnitude of change. We rigorously tested five models of working memory limitations, each consisting of an encoding stage and a decision stage. The encoding stage differed between the five models: the original item-limit model [2], [15]–[16], two recent variants [9], and two continuous-resource models, one with equal precision for all items [20], [23], and one with item-to-item and trial-to-trial variability in precision [13], [33]. The decision stage was Bayesian for every model. To anticipate our results, we find that variable precision coupled with Bayesian inference provides a highly accurate account of human working memory performance across change magnitudes, set sizes, and feature dimensions, and far outperforms models that postulate an item limit.

## Results

### Theory

**Figure 1:**
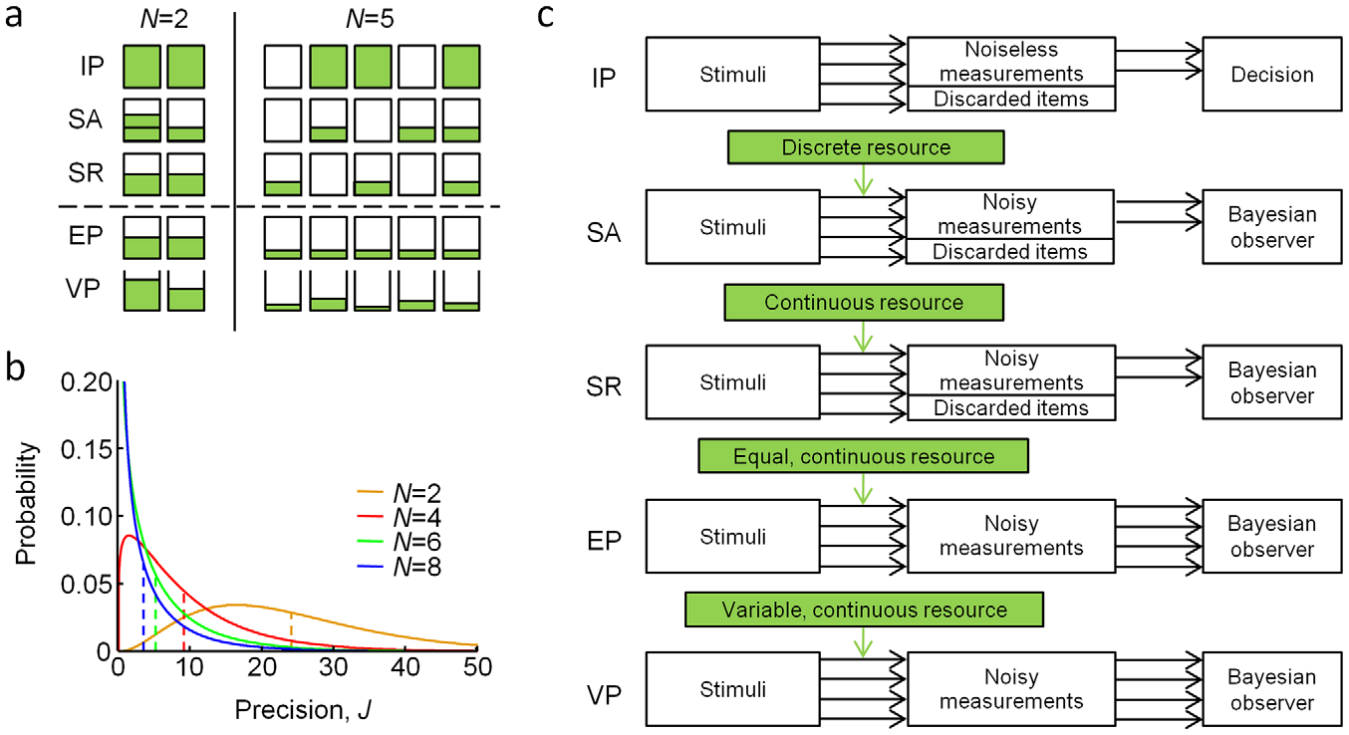
Models of change detection. Infinite-precision item limit (IP), slots plus averaging (SA), slots plus resources (SR), equal precision (EP), and variable precision (VP). The first three are item-limit models, the last two continuous-resource models. (**a**) Illustration of resource allocation in the models at set sizes 2 and 5, with a capacity of 3 slots/chunks for IP, SA, and SR. The VP model is distinct from the other models in that the amount of resource varies on a continuum without a hard upper bound. (**b**) Probability density functions over encoding precision in the VP model, for four set sizes. Parameters were taken from the best fit to the data of one human subject. Mean precision, indicated by a dashed line, is inversely proportional to set size. In the EP model, these distributions would be infinitely sharp (delta functions). (**c**) Decision process during change detection for each of the five models.

We model a task in which the observer is presented with two displays, each containing *N* oriented stimuli and separated in time by a delay period. On each trial, there is a 50% probability that one stimulus changes orientation between the first and the second display. The change can be of any magnitude. Observers report whether or not a change occurred. We tested five models of this task, which differ in the way they conceptualize what memory resource consists of and how it is distributed across items (Fig. 1a).

#### Infinite-precision item-limit model

In the infinite-precision (IP) item-limit model, the oldest item-limit model [2], [8], [15]–[16] and often called the “limited-capacity” or simply the “item-limit” model, memorized items are stored in one of *K* available “slots”. *K* is called the capacity. Each slot can hold exactly one item. The memory of a stored item is perfect (“infinite precision”). If *N*≤*K*, all items from the first display are stored. If *N*>*K*, the observer memorizes *K* randomly chosen items from the first display. When a change occurs among the memorized items, the observer responds “change” with probability 1−*ε*. When no change occurs among the memorized items, the observer responds “change” with a guessing probability *g*.

#### Precision and noise

All models other than the IP model assume that the observer's measurement of each stimulus is corrupted by noise. We model the measurement *x* of a stimulus *θ* as being drawn from a Von Mises (circular normal) distribution centered at *θ*:
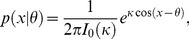
(1)where *κ* is called the concentration parameter and *I*_0_ is the modified Bessel function of the first kind of order 0. (For convenience, we remap all orientations from [−π/2, π/2) to [−π, π).)

In all models with measurement noise, we identify memory resource with Fisher information, *J*(*θ*) [34]. The reasons for this choice are threefold [13]. First, regardless of the functional form of the distribution of the internal representation of a stimulus (in our formalism, of the scalar measurement), Fisher information determines the best possible performance of any estimator through the Cramér-Rao bound [34], of which a version on a circular space exists [35]. Second, when the measurement distribution is Gaussian, Fisher information is equal to the inverse variance, 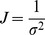, which is, up to an irrelevant proportionality constant, the same relationship one would obtain by regarding resource as a collection of discrete observations or samples [20], [23]. Third, when neural variability is Poisson-like, Fisher information is proportional to the gain of the neural population [36]–[38], and therefore the choice of Fisher information is consistent with regarding neural activity as resource [13]. We will routinely refer to Fisher information as precision. For the circular measurement distribution in Eq. (1), Fisher information is related to *κ* through 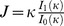 [13], [33], where *I*_1_(*κ*) is the modified Bessel function of the first kind of order 1.

#### Slots-plus-averaging model

The SA model [9] is an item-limit model in which *K* discrete, indivisible chunks of resource are allocated to items. When *N*>*K*, *K* randomly chosen items receive a chunk and are encoded; the remaining *N*−*K* items are not memorized. When *N*≤*K*, chunks are distributed as evenly as possible over all items. For example, if *K* = 4 and *N* = 3, two items receive one chunk and one receives two. Resource per item, *J*, is proportional to the number of chunks allocated to it, denoted *S*: *J* = *SJ*_s_, where *J*_s_ is the Fisher information corresponding to one chunk.

#### Slots-plus-resources model

The slots-plus-resources (SR) model [9] is identical to the SA model, except that resource does not come in discrete chunks but is a continuous quantity. When *N*≤*K*, all items are encoded with precision *J* = *J*_1_/*N*, where *J*_1_ is the Fisher information for a single item. When *N*>*K*, *K* randomly chosen items are encoded with precision *J* = *J*_1_/*K* and the remaining *N*−*K* items are not memorized. Related but less quantitative ideas have been proposed by Alvarez and Cavanagh [14] and by Awh and colleagues [7], [18].

#### Equal-precision model

According to the equal-precision (EP) model [10]–[11], [20], [23], precision is a continuous quantity that is equally divided over all items. Versions of this model have been tested before on change detection data [8], [10], [39]. If the total amount of memory precision were fixed across trials, we would expect an inverse proportionality between *J* and set size. However, there is no strong justification for this assumption, we allow for a more flexible relationship by using a power-law function, *J* = *J*_1_ *N^α^*.

#### Variable-precision model

In the variable-precision (VP) model [13], encoding precision is variable across items and trials, and average encoding precision depends on set size. We model variability in precision by drawing *J* from a gamma distribution with mean 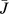 and scale parameter *τ* (Fig. 1b). The gamma distribution is a flexible, two-parameter family of distributions on the positive real line. The process by which a measurement *x* is generated in the VP model is thus doubly stochastic: *x* is drawn randomly from a Von Mises distribution with a given precision, while precision itself is stochastic. Analogous to *J* in the EP model, we model the relationship between 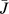 and set size using a power law function, 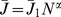.

#### Bayesian inference

In the models with noise (SA, SR, EP, VP), the observer decides whether or not a change occurred (denoted by *C* = 1 and *C* = 0) based on the noisy measurements in both displays (Fig. 1c). We use *x_i_* and *y_i_* to denote the noisy measurements at the *i* ^th^ location in the first and second displays, and *κ_x,i_* and *κ_y,i_* are their respective concentration parameters (see Eq. (1)). Due to the noise, the measurements of any one item will always differ between displays, even if the underlying stimulus value remains unchanged. Thus, also on no-change trials, the observer is confronted with two non-identical sets of measurements, making the inference problem difficult. While the noise precludes perfect performance, the observer still has a best possible strategy available, namely Bayesian MAP estimation. This strategy consists of computing, on each trial, the probability of a change based on the measurements, *p*(*C* = 1|**x**,**y**), where **x** and **y** are the vectors of measurements {*x_i_*} and {*y_i_*}, respectively. The observer then responds “change” if this probability exceeds 0.5, or in other words, when
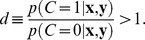



Making use of the statistical structure of the task (Fig. S1), the posterior ratio *d* can be evaluated to
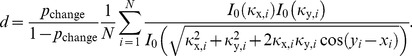
(2)(see Text S1 and [40]). Here, *p*_change_ is the prior probability that a change occurred. This decision rule automatically models errors arising in the comparison operation [1], [18], [29]–[30]: the difference *y_i_*−*x_i_* is noisy, so that even when a change is absent, it might by chance be large, and even when a change is present, it might by chance be small.

In an earlier paper [40], we examined suboptimal alternative decision rules. A plausible one would be a “threshold” rule, according to which the observer compares the largest difference between measurements at the same location in the two displays to a fixed criterion. If the difference exceeds the criterion, the observer reports that a change occurred. We proposed this “maximum-absolute-difference” rule in our earlier continuous-resource treatment of change detection [10], but a comparison against the optimal rule showed it to be inadequate [40].

Another suboptimal strategy that deserves attention is probability matching or sampling [41]–[42]. Under this strategy, the observer computes the Bayesian posterior *p*(*C* = 1|**x**,**y**), but instead of reporting a change (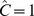) when this probability exceeds 0.5, reports a change with probability
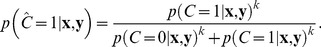
(3)


When *k* = 0, probability matching amounts to random guessing; when *k*→∞, it reduces to MAP estimation. Thus, probability matching consists of a family of stochastic decision rules interpolating between MAP estimation and guessing. Probability matching turns out to be very similar to a modification of MAP estimation we considered in [40], namely adding zero-mean Gaussian noise to the logarithm of the decision variable in Eq. (2). To see this, we rewrite Eq. (3) as
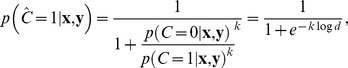
which is the logistic function with argument log *d*. On the other hand, adding zero-mean Gaussian noise *η* with standard deviation *σ_η_* to log *d* gives
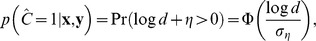
where Φ is the cumulative of the standard normal distribution. It is easy to verify that the logistic function and the cumulative normal distribution are close approximations of each other (with a one-to-one relation between *k* and *σ_η_*), showing that both forms of suboptimality are very similar. Since an equal-precision model augmented with Gaussian decision noise far underperformed the variable-precision model [40], human data are unlikely to be explained by such decision noise (or equivalently by probability matching) in the absence of variable precision in the encoding stage. It is, however, possible that decision noise is present in addition to variability in encoding precision, but this would not invalidate our conclusions. Therefore, in the present paper, we will only examine the optimal Bayesian decision rule.

#### Free parameters

The IP, SA, SR, and EP models each have 3 free parameters, and the VP model has 4.

### Experiment: orientation change detection

**Figure 2:**
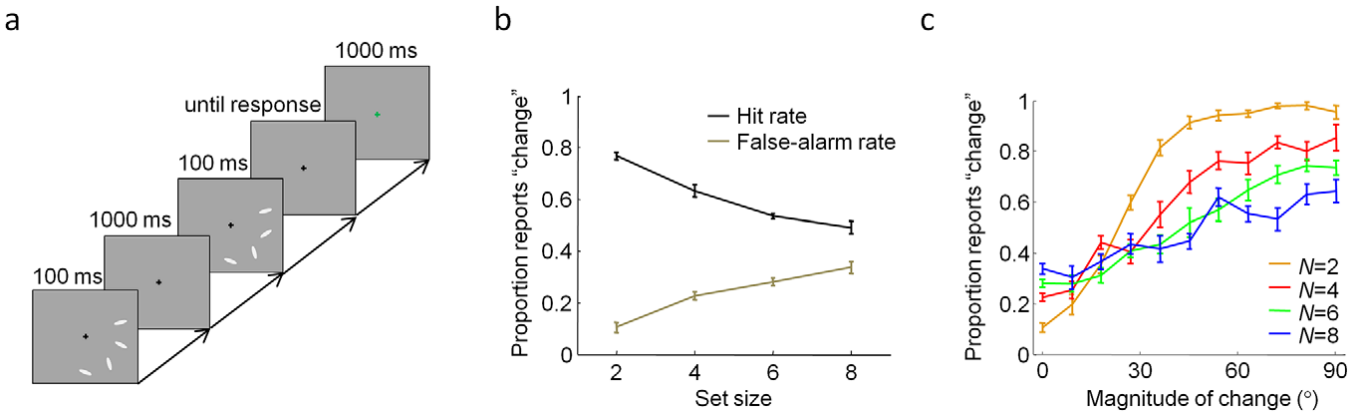
Orientation change detection. (**a**) Observers reported whether one of the orientations changed between the first and second displays. (**b**) Hit and false-alarm rates as a function of set size. (**c**) Psychometric curves, showing the proportion of “change” reports as a function of the magnitude of change, for each set size (mean ± s.e.m across subjects). Magnitude of change was binned into 9° bins. The first point on each curve (at 0°) contains all trials in which no change occurred, and thus represents the false-alarm rate. Using the standard formula for *K* would return different estimates for different change magnitudes.

We conducted an orientation change detection task in which we manipulated both set size and change magnitude (Fig. 2a). Consistent with earlier studies (e.g. [10], [15], [17]), we found that the ability of observers to detect a change decreased with set size, with hit rate *H* monotonically decreasing and false-alarm rate *F* monotonically increasing (Fig. 2b). Effects of set size were significant (repeated-measures ANOVA; hit rate: *F*(3,27) = 52.8, *p*<0.001; false alarm rate: *F*(3,27) = 82.0, *p*<0.001). The increase in *F* is inconsistent with the IP model, as this model would predict no dependence.

For a more detailed representation of the data, we binned magnitude of change on change trials into 10 bins (Fig. 2c). All no-change trials had magnitude 0 and sat in a separate bin. These psychometric curves clearly show that the probability of reporting a change increases with change magnitude at every set size (*p*<0.001). From Fig. 2c we could, in principle, compute a naïve estimate of memory capacity using the well-known formula from the IP model, *K* = *N*(*H*−*F*)/(1−*F*) [16]. However, since *H* depends on the magnitude of change, the estimated *K* would depend on the magnitude of change as well, contradicting the basic premise of a fixed capacity. For example, at set size 6, for change magnitudes between 0° and 9°, Cowan's formula would estimate *K* at exactly zero (no items retained at all), while for magnitudes between 81° and 90°, it would estimate *K* at 3.8, with a nearly linear increase in between. This serves as a first indication that the IP model in general and this formula in particular are wrong.

#### Model fits

**Figure 3:**
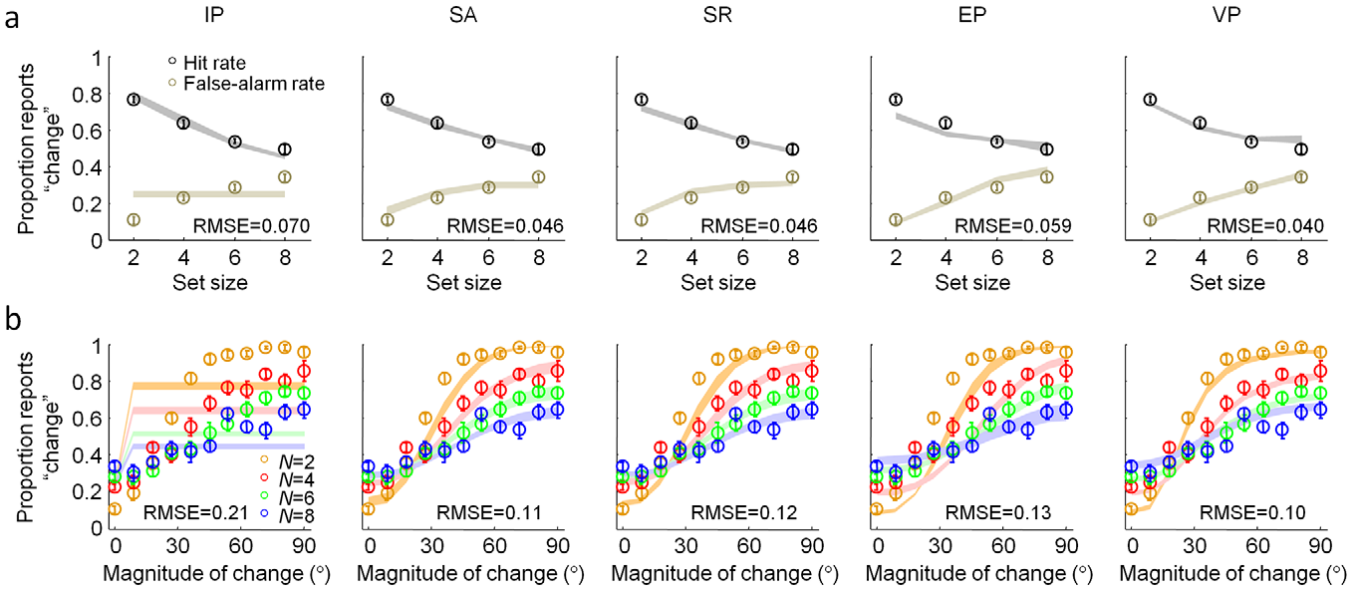
Comparing models on summary statistics. (**a**) Model fits to the hit and false-alarm rates. (**b**) Model fits to the psychometric curves. Shaded areas represent ±1 s.e.m. in the model. For the IL model, a change of magnitude 0 has a separate proportion reports “change”, equal to the false-alarm rate shown in (a). In each plot, the root mean square error between the means of data and model is given.

We fitted all models using maximum-likelihood estimation, for each subject separately (see Text S1). Mean and standard error of all parameters of all models are shown in Table 1. The values of capacity *K* in the IP, SA, and SR models were 3.10±0.28, 4.30±0.47, and 4.30±0.42, respectively (mean and s.e.m.), in line with earlier studies [7]–[9], [15]–[18]. Using the maximum-likelihood estimates of the parameters, we obtained hit rates, false-alarm rates, and psychometric curves for each model and each subject (Fig. 3).

**Table 1:** Fitted parameter ranges and estimates.

		Experiment 1 estimates		Tested range	
Model	Parameter	Mean	s.e.m.	Min	Max
**IP**	*K*	3.10	0.28	1	8
	*ε*	0.220	0.021	0	1
	*G*	0.247	0.016	0	0.5
**SA**	*J*_s_	3.94	0.58	1	40
	*K*	4.30	0.47	1	8
	*p*_change_	0.584	0.020	0.2	0.8
**SR**	*J*_1_	14.2	1.7	1	60
	*K*	4.30	0.42	1	8
	*p*_change_	0.574	0.019	0.2	0.8
**EP**	*J*_1_	20.3	3.6	1	60
	*α*	−1.28	0.11	−2	0
	*p*_change_	0.492	0.007	0.2	0.8
**VP**	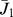	53.1	6.1	5	300
	*τ*	31.2	8.9	5	300
	*α*	−0.88	0.08	−2	0
	*p*_change_	0.532	0.005	0.2	0.8

Mean and standard error of the maximum-likelihood estimates and tested ranges of model parameters for Experiment 1 (orientation change detection).

Hit and false-alarm rates were best described by the VP model, per root-mean-square error (RMSE) of the subject means (0.040), followed by the SA and SR models (both 0.046), the equal-precision (EP) model (0.059), and the IP model (0.070). The same order was found for the psychometric curves (RMSE: 0.10 for VP, 0.11 for SA, 0.12 for SR, 0.13 for EP, and 0.21 for IP). The IP model predicts that performance is independent of magnitude of change and is therefore easy to rule out.

#### Bayesian model comparison

**Figure 4:**
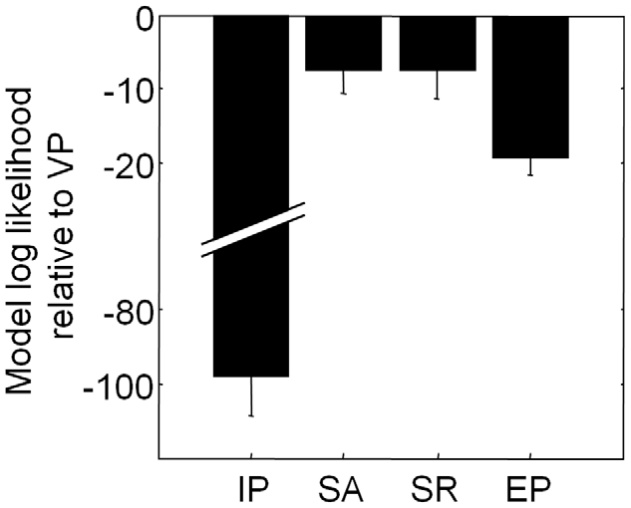
Bayesian model comparison. Model log likelihood of each model minus that of the VP model (mean ± s.e.m.). A value of −*x* means that the data are *e^x^* times more probable under the VP model.

The RMS errors reported so far are rather arbitrary descriptive statistics. To compare the models in a more principled (though less visualizable) fashion, we performed Bayesian model comparison, also called Bayes factors [43]–[44] (see Text S1). This method returns the likelihood of each model given the data and has three desirable properties: it uses all data instead of only a subset (like cross-validation would) or summary; it does not solely rely on point estimates of the parameters but integrates over parameter space, thereby accounting for the model's robustness against variations in the parameters; it automatically incorporates a correction for the number of free parameters. We found that the log likelihood of the VP model exceeds that of the IP, SA, SR, and EP models by 97±11, 7.2±3.5, 7.4±3.7, and 19±3, respectively (Fig. 4). This constitutes strong evidence in favor of the VP model, for example according to Jeffreys' scale [45]. Based on our data, we can convincingly rule out the three item-limit models (IP, SA, and SR) as well as the equal-precision (EP) model, as descriptions of human change detection behavior.

#### Apparent guessing as an epiphenomenon

In the delayed-estimation paradigm of working memory [10], data consist of subject's estimates of a memorized stimulus on a continuous space. Zhang and Luck [9] analyzed the histograms of estimation errors in this task by fitting a mixture of a uniform distribution (allegedly representing guesses) and a Von Mises distribution (allegedly representing true estimates of the target stimulus). They suggested that the mixture proportion of the uniform distribution represents the rate at which subjects guess randomly, and interpreted its increase with set size as evidence for a fixed limit on the number of remembered items. However, Van den Berg et al. [13] later showed that the variable-precision model reproduces the increase of the mixture proportion of the uniform distribution with set size well, even though the model does not contain any pure guessing. They suggested that the guesses reported in the mixture analysis were merely “apparent guesses”.

**Figure 5:**
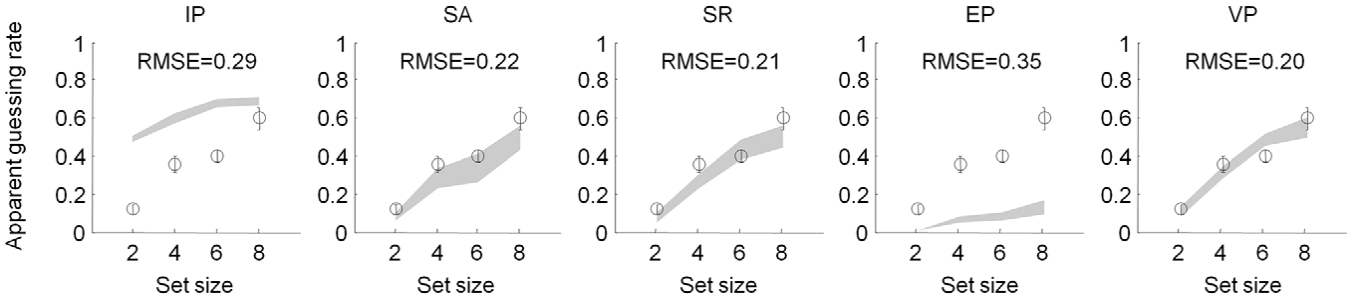
Apparent guessing analysis. Apparent guessing rate as a function of set size as obtained from subject data (circles and error bars) and synthetic data generated by each model (shaded areas). Even though the VP model does not contain any “true” guesses, it still accounts best for the apparent guessing rate.

We perform an analogous analysis for change detection here. We fitted, at each set size separately, a model in which subjects guess on a certain proportion of trials, and on other trials, respond like an EP observer. Free parameters, at each set size separately, are the guessing parameter, which we call apparent guessing rate (AGR), and the precision parameter of the EP observer. We found that AGR was significantly different from zero at every set size (*t*(9)>4.5, *p*<0.001) and increased with set size (Fig. 5; repeated-measures ANOVA, main effect of set size: *F*(3,27) = 21.1, *p*<0.001), reaching as much as 0.60±0.06 at set size 8.

We then examined how well each of our five models can reproduce the increase of AGR. To do so, we computed AGR from synthetic data generated using each model, using maximum-likelihood estimates of the parameters as obtained from the subjects' data. We found that the VP model – which does not contain any actual guessing – reproduces the apparent guessing rate better than the other models (Fig. 5; RMSE = 0.20 for VP). This means that the apparent presence of guessing does not imply that visual working memory is item-limited.

How the VP model can reproduce apparent guessing can be understood as follows. In the VP model, the distribution of precision is typically broad and includes a lot of small values, especially at larger set sizes (Fig. 1b). The EP model augmented with set size-dependent guessing would approximate this broad distribution by one consisting of two spikes of probability, one at a nonzero, fixed precision and one at zero precision. To mimic the VP precision distribution, the weight of the spike at zero must increase with set size, leading to an increase of AGR with set size. In sum, variability in precision produces apparent guessing as an epiphenomenon, a finding that is consistent with our results in the delayed-estimation task [13].

#### Generalization

To assess the generality of our results, we repeated the orientation change detection experiment with color stimuli and found consistent results (see Figs. S2, S3, S4, S5 and Text S1). Specifically, in Bayesian model comparison, the VP model outperforms all other models by log likelihood differences of at least 48.4±8.2, which constitutes further evidence against an item limit.

## Discussion

### Implications for working memory

Five models of visual working memory limitations have been proposed in the literature. Here, we tested all five using a change detection paradigm. Although change detection has been investigated extensively, several of the models had never been applied to this task and no previous study had compared all models. Compared to previous studies, our use of a continuous stimulus variable and changes drawn from a wide range of magnitudes enhanced our ability to tell apart the model predictions. Our results suggest that working memory resource is continuous and variable and do not support the notion of an item limit.

The variable-precision model of change detection connects a continuous-resource encoding model of working memory [13] with a Bayesian model for decision-making in change detection [33]. This improves on two related change detection studies that advocated for continuous resources. Wilken and Ma [10] introduced the concept of continuous resources, but only compared an EP model with a suboptimal decision rule to the IP model. Although the EP model won in this comparison, the more recent item-limit models (SA and SR) had not yet been proposed at that time. Our present results show that the SA and SR models are improvements over both the EP and IP models, but lose to the VP model. In a more recent study, we compared different variants of the Bayesian model of the decision process and found that the optimal decision rule outperformed suboptimal ones [33], but we did not vary set size or compare different models of working memory. Other tasks, such as change localization [13], visual search [21], [23], and multiple-object tracking [19], [46], can also be conceptualized using a resource-limited front end conjoined with a Bayesian-observer back end. Whether such a conceptualization will survive a deeper understanding of resource limitations remains to be seen.

It is instructive to consider each model in terms of the distribution over precision that it postulates for a given set size. In the IP model, this distribution has mass at infinity and, depending on set size, also at zero. In the SA and SR models, probability mass resides, depending on set size, at one or two nonzero values, or at zero and one nonzero value. The EP model has probability mass only at one nonzero value. The VP model is the only model considered that assigns probability to a broad, continuous range of precision values. Roughly speaking, the more values of precision a model allows, the better it seems to fit. Although we assumed in the VP model that precision follows a gamma distribution, it is possible that a different continuous distribution can describe variability in precision better. However, the amount of data needed to distinguish different continuous precision distributions using psychophysics only might be prohibitive.

Work by Rouder et al. used a change detection task to compare a continuous-resource model based on signal detection theory to a variant of the IP model [8]. Manipulating bias, they measured receiver-operating characteristics (ROCs). The IP variant predicted straight-line ROCs, whereas the continuous-resource model predicted regular ROCs (i.e., passing through the origin). Unfortunately, each of the ROCs they measured contained only three points, and therefore the models were very difficult to distinguish. We ourselves, in an earlier study, had collected five-point ROCs using confidence ratings, allowing for an easier distinction between different ROC types; there, we found that the ROCs were regular [10], in support of a continuous-resource model. A difference between the Rouder study and our current study is that Rouder et al. used ten distinct colors instead of a one-dimensional continuum; this again has the disadvantage of missing the stimulus regime in which the signal-to-noise ratio is low. Moreover, the decision process in their continuous-resource model was not optimal; an optimal observer would utilize knowledge of the distribution of the stimuli and change magnitudes used in the experiment. It is likely that the optimal decision rule would have described human behavior in Rouder et al.'s experiment better than an ad-hoc suboptimal rule [33]. Finally, Rouder et al. did not consider variability in precision. In short, our current study does not contradict the results of Rouder et al., but offers a more plausible continuous-resource model and tests all models over a broader range of experimental conditions.

The notion of an item limit on the one hand and continuous or variable resources on the other hand are not mutually exclusive. In the SR model, for example, a continuous resource is split among a limited number of items. Although this model was not the best in the present study, many other “hybrid” models can be conceived – such as a VP model augmented with an item limit, or an IP or SA model with variable capacity [47]–[48] – and testing them is an important direction for future work. Our results, however, establish the VP model as the standard against which any new model of change detection should be compared.

### Neural implications

The neural basis of working memory limitations is unknown. In the variable-precision model, encoding precision is the central concept, raising the question which neural quantity corresponds to encoding precision. We hypothesize that precision relates to neural gain, according to the reasoning laid out in previous work [13], [19], [33]. To summarize, gain translates directly to precision in sensory population codes [49], increased gain correlates with increased attention [50], and high gain is energetically costly [51], potentially bringing encoding precision down as set size increases. The variable-precision model predicts that the gain associated with the encoding of each item exhibits large fluctuations across items and trials. There is initial neurophysiological support for this prediction [52]–[53]. Furthermore, if gain is variable, then spiking activity originates from a doubly stochastic process: spiking is stochastic for a given of value of gain, while gain is stochastic itself. Recent evidence points in this direction [54]–[55], although formal model comparison remains to be done. The variable-precision model also predicts that gain on average decreases with increasing set size. We proposed in earlier work that this could be realized mechanistically by divisive normalization [19]. Divisive normalization could act on the gains of the input populations by approximately dividing each gain by the sum of the gains across all locations raised to some power [56]. When set size is larger, the division would be by a larger number, resulting in a post-normalization gain that decreases with set size. A spiking neural network implementation of aspects of continuous-resource models was proposed recently [57]. Taken together, the variable-precision model has plausible neural underpinnings.

Our results have far-reaching implications for neural studies of working memory limitations. Throughout the field, taking a fixed item limit for granted has been the norm, and many studies have focused on finding its neural correlates [12], [58]. Even if we restrict ourselves to change detection only, a fixed item limit has been assumed by studies that used fMRI [59]–[65], EEG [66]–[72], MEG [67], [72]–[73], voxel-based morphometry [74], TMS [68], [75], lesion patients [76], and computational models [77]–[78]. Our present results undermine the theoretical basis of all these studies. Neural studies that questioned the item-limit model or attempted to correlate neural measures with parameters in a continuous-resource model have been rare [27], [57]. Perhaps, this is because no continuous-resource model has so far been perceived as compelling. The variable-precision model remedies this situation and might inspire a new generation of neural studies.

## Materials and Methods

### Stimuli

Stimuli were displayed on a 21″ LCD monitor at a viewing distance of approximately 60 cm. Stimuli were oriented ellipses with minor and major axes of 0.41 and 0.94 degrees of visual angle (deg), respectively. On each trial, ellipse centers were chosen by placing one at a random location on an imaginary circle of radius 7 deg around the screen center, placing the next one 45° counterclockwise from the first along the circle, etc., until all ellipses had been placed. Set size was 2, 4, 6, or 8. Each ellipse position was jittered by a random amount between −0.3 and 0.3 deg in both *x*- and *y*-directions to reduce the probability of orientation alignments between items. Stimulus and background luminances were 95.7 and 33.1 cd/m^2^, respectively.

### Participants

Ten observers participated (4 female, 6 male; 3 authors). All were between 20 and 35 years old, had normal or corrected-to-normal vision, and gave informed consent.

### Procedure

On each trial, the first stimulus display was presented for 117 ms, followed by a delay period (1000 ms) and a second stimulus display (117 ms). In the first display, set size was chosen randomly and the orientation of each item was drawn independently from a uniform distribution over all possible orientations. The second display was identical to the first, except that there was a 50% chance that one of the ellipses had changed its orientation by an angle drawn from a uniform distribution over all possible orientations. The ellipse centers in the second screen were jittered independently from those in the first. Following the second display, the observer pressed a key to indicate whether there was a change between the first and second displays. A correct response caused the fixation cross to turn green and an incorrect response caused it to turn red. During the instruction phase, observers were informed in lay terms about the distributions from which the stimuli were drawn (e.g., “The change is equally likely to be of any magnitude.”). Each observer completed three sessions of 600 trials each, with each session on a separate day, for a total of 1800 trials. There were timed breaks after every 100 trials. During each break, the screen displayed the observer's cumulative percentage correct.

### Model fitting and model comparison

Methods for model fitting and model comparison are described in the Text S1.

## Supporting Information

Figure S1 **Generative model.** The generative model shows the relevant variables in the change detection task and the statistical dependencies between them. *C*: change occurrence (0 or 1); Δ: magnitude of change; **Δ**: vector of change magnitudes at all locations; **θ** and **φ**: vectors of stimuli in the first and second displays, respectively; **x** and **y**: vectors of measurements in the first and second displays, respectively.
(TIF)

Figure S2 **Color change detection.** Observers reported whether one of the colors changed between the first and second displays.
(TIF)

Figure S3 **Color change detection: summary statistics and model fits.** (**a**) Model fits to the hit and false-alarm rates. (**b**) Model fits to the psychometric curves. Shaded areas represent ±1 s.e.m. in the model. For the IL model, a change of magnitude 0 has a separate proportion reports “change”, equal to the false-alarm rate shown in (a). In each plot, the root mean square error between the means of data and model is given.
(TIF)

Figure S4 **Color change detection: Bayesian model comparison.** Model log likelihood of each model minus that of the VP model (mean ± s.e.m.). A value of −*x* means that the data are *e^x^* times more probable under the VP model.
(TIF)

Figure S5 **Color change detection: apparent guessing analysis.** Apparent guessing rate as a function of set size as obtained from subject data (circles and error bars) and synthetic data generated by each model (shaded areas). Even though the VP model does not contain any “true” guesses, it still accounts best for the apparent guessing rate.
(TIF)

Table S1 **Mean and standard error of the maximum-likelihood estimates and tested ranges of model parameters for Experiment 2 (color change detection).**
(DOCX)

Text S1 **Supporting text.** Detailed derivation of Bayesian decision rule and explanation of model fitting and comparison methods. Explanation of color change detection experiment and results.
(DOCX)
