# Supplementary figures and images for: No Evidence for an Item Limit in Change Detection
Source: PLoS Comput Biol. 2013 Feb 28;9(2):e1002927. doi: 10.1371/journal.pcbi.1002927 (PMC3585403; doi:10.1371/journal.pcbi.1002927)

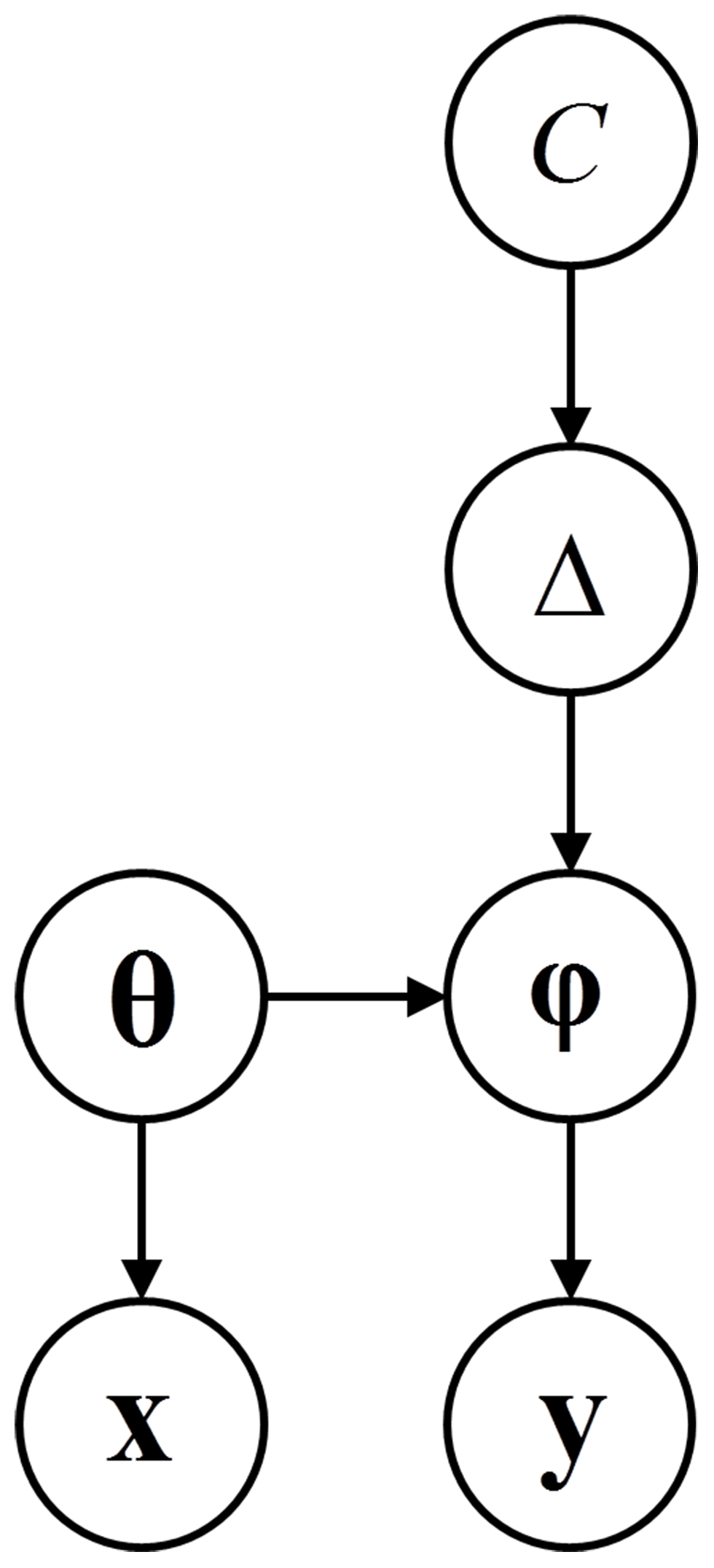

Supplement: Figure S1 — Generative model. The generative model shows the relevant variables in the change detection task and the statistical dependencies between them. C: change occurrence (0 or 1); Δ: magnitude of change; Δ: vector of change magnitudes at all locations; θ and φ: vectors of stimuli in the first and second displays, respectively; x and y: vectors of measurements in the first and second displays, respectively. (TIF) [file pcbi.1002927.s001.tif]

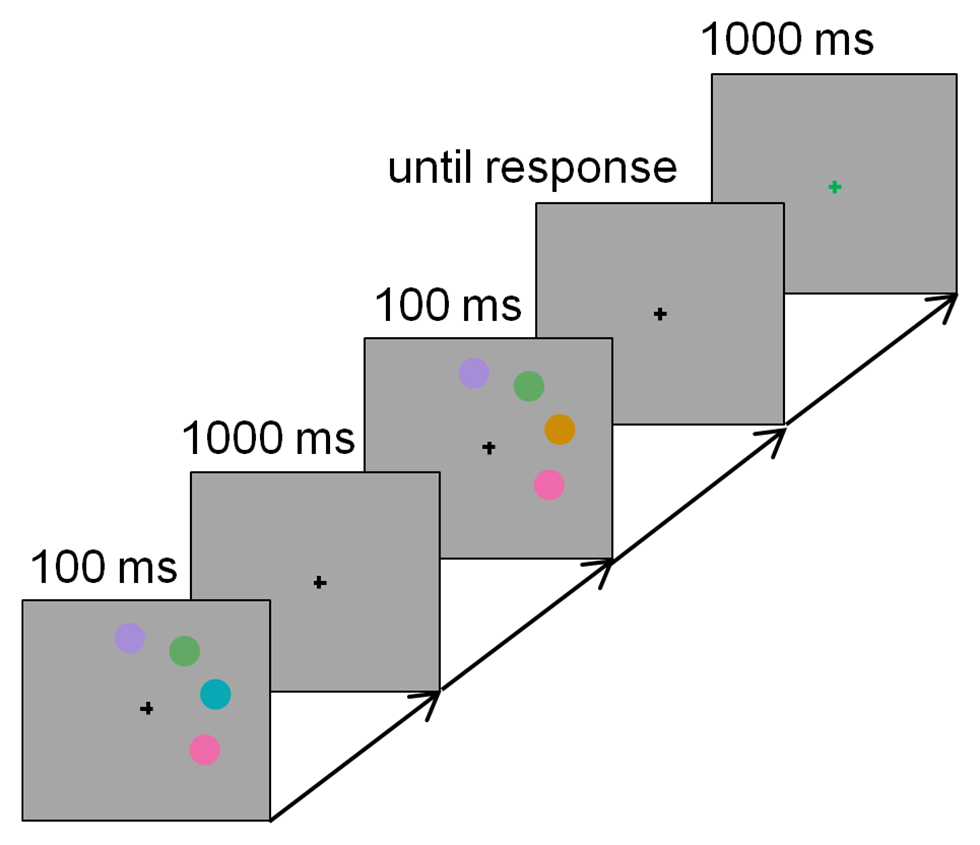

Supplement: Figure S2 — Color change detection. Observers reported whether one of the colors changed between the first and second displays. (TIF) [file pcbi.1002927.s002.tif]

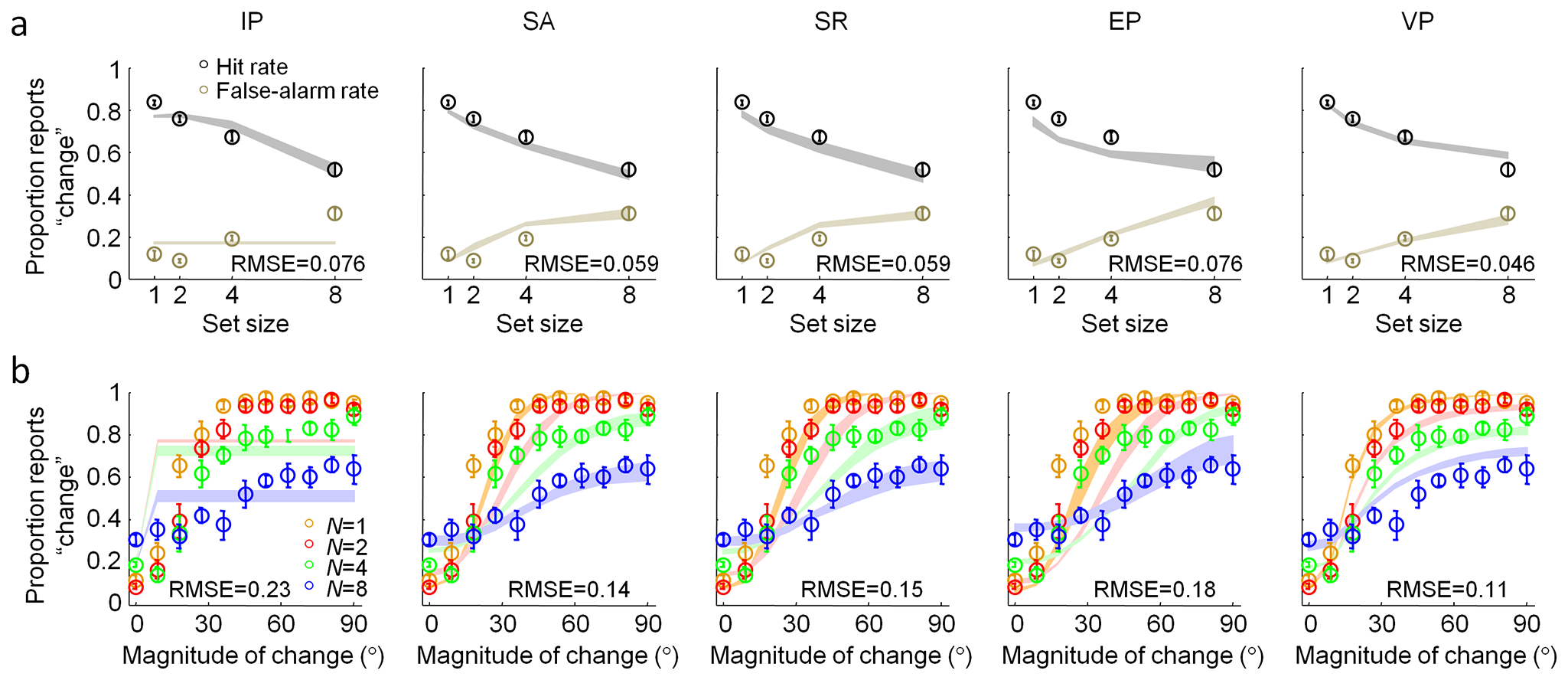

Supplement: Figure S3 — Color change detection: summary statistics and model fits. (a) Model fits to the hit and false-alarm rates. (b) Model fits to the psychometric curves. Shaded areas represent ±1 s.e.m. in the model. For the IL model, a change of magnitude 0 has a separate proportion reports “change”, equal to the false-alarm rate shown in (a). In each plot, the root mean square error between the means of data and model is given. (TIF) [file pcbi.1002927.s003.tif]
