# Supplementary material for: No Evidence for an Item Limit in Change Detection
Source: PLoS Comput Biol. 2013 Feb 28;9(2):e1002927. doi: 10.1371/journal.pcbi.1002927 (PMC3585403; doi:10.1371/journal.pcbi.1002927)
Supplement: Table S1 — Mean and standard error of the maximum-likelihood estimates and tested ranges of model parameters for Experiment 2 (color change detection). (DOCX) [file pcbi.1002927.s006.docx]

**Table S1.** Mean and standard error of the maximum-likelihood estimates and tested ranges of model parameters for Experiment 2 (color change detection).

|  | | **Experiment 2 estimates** | | **Tested range** | |
| --- | --- | --- | --- | --- | --- |
| **Model** | **Parameter** | **Mean** | **S.e.m.** | **Min** | **Max** |
| IL | *K* | 4.57 | 0.43 | 1 | 8 |
|  | *ε* | 0.229 | 0.009 | 0 | 1 |
|  | *G* | 0.172 | 0.005 | 0 | 0.5 |
| SA | *J*_1_ | 3.97 | 0.72 | 1 | 40 |
|  | *K* | 4.14 | 0.59 | 1 | 8 |
|  | *p*_change_ | 0.591 | 0.016 | 0.2 | 0.8 |
| SR | *J*_1_ | 13.8 | 2.0 | 1 | 60 |
|  | *K* | 4.14 | 0.46 | 1 | 8 |
|  | *p*_change_ | 0.574 | 0.019 | 0.2 | 0.8 |
| EP | *J*_1_ | 13.6 | 4.1 | 1 | 60 |
|  | α | -0.914 | 0.195 | -2 | 0 |
|  | *p*_change_ | 0.506 | 0.008 | 0.2 | 0.8 |
| VP | $\bar{J}_{1}$ | 111 | 14.0 | 5 | 300 |
|  | *τ* | 163 | 35 | 5 | 300 |
|  | *α* | -0.471 | 0.042 | -2 | 0 |
|  | *p*_change_ | 0.557 | 0.005 | 0.2 | 0.8 |
