# Supplementary material for: No Evidence for an Item Limit in Change Detection
Source: PLoS Comput Biol. 2013 Feb 28;9(2):e1002927. doi: 10.1371/journal.pcbi.1002927 (PMC3585403; doi:10.1371/journal.pcbi.1002927)
Supplement: Text S1 — Supporting text. Detailed derivation of Bayesian decision rule and explanation of model fitting and comparison methods. Explanation of color change detection experiment and results. (DOCX) [file pcbi.1002927.s007.docx]

**No evidence for an item limit in change detection**

Shaiyan Keshvari, Ronald van den Berg, Wei Ji Ma

# Supporting text

The Bayesian decision process in change detection

The first step in Bayesian modeling of a perceptual task is to specify the statistical structure of the task, also called the generative model (Fig. S1). We denote change occurrence by *C*, a binary variable taking values 0 and 1 (0: no change; 1: change). Each of the *N* stimuli in the first display, denoted by a vector **θ**=(*θ*_1_,…,*θ_N_*), takes values on the circle. They are drawn independently from a uniform distribution over the stimulus space, so that *p*(**θ**)=1/(2*π*)*^N^*. All locations are equally likely to contain the change. The magnitude of change, denoted by Δ, is drawn from a uniform distribution, *p*(Δ)=1/(2*π*). The *N* stimuli in the second display, denoted by **ϕ**=(*ϕ*_1_,…,*ϕ_N_*), are equal to **θ**, except for one randomly chosen entry when *C*=1. Finally, the measurements in the first and second displays, **x**=(*x*_1_,…,*x_N_*) and **y**=(*y*_1_,…,*y_N_*), respectively, are drawn from independent Von Mises distributions with corresponding concentration parameters **κ**_x_=(*κ*_x,1_,…, *κ*_x_*_,N_*) and **κ**_y_=(*κ*_y,1_,…, *κ*_y_*_,N_*):

This allows for the possibility that the concentration parameter of the noise at a given location is different between the first and the second displays. In the variable-precision model, this will in general be the case, since these values are modeled as being drawn independently.

Now we are ready to work out the observer’s inference process. Bayesian inference amounts to computing the probability of *C* given the noisy measurements in the first and second displays on a given trial, **x** and **y**. The Bayesian decision variable *d* is the ratio between the probability that a change occurred and the probability that no change occurred, given **x** and **y**:

$$d=\frac{p\left(C=1|\mathbf{x},\mathbf{y}\right)}{p\left(C=0|\mathbf{x},\mathbf{y}\right)}=\frac{p\left(\mathbf{x},\mathbf{y}|C=1\right)p\left(C=1\right)}{p\left(\mathbf{x},\mathbf{y}|C=0\right)p\left(C=0\right)},$$

where *p*(*C*) is the prior probability of change occurrence. *d* is called the *posterior ratio* of change occurrence and reflects the observer’s belief about the occurrence of a change on one trial. To compute *d*, we need *p*(**x**,**y**|*C*), the distribution of the measurements in either the no-change or the change conditions. This distribution is obtained by marginalizing the joint distribution of all variables over Δ, **θ**, and **ϕ**. The result is

$$p\left(\mathbf{x},\mathbf{y}|C\right)=\left(\frac{1}{2\pi}\right)^{N+1}\frac{1}{N}\sum_{i=1}^{N} \left(\prod_{j\neq i}^{N} \int p\left(x_{j}|\theta_{j}\right)p\left(y_{j}|\theta_{j}\right)d\theta_{j}\right)\iint p\left(x_{i}|\theta_{i}\right)p\left(y_{i}|\theta_{i}+C\Delta\right)d\theta_{i}d\Delta.$$

The sum over *i* runs over the *N* possible locations of the change. In Eq. , *p*(*x_i_*|*θ_i_*) and *p*(*y_i_*|*ϕ_i_*) are interpreted as likelihood functions over the stimulus – single-trial quantities that reflect the observer’s belief about the stimulus (*θ_i_* or *ϕ_i_*) based on the measurement on that trial (*x_i_* or *y_i_*). The width of the stimulus likelihood function reflects the observer’s uncertainty about the stimulus.

Twice substituting Eq. in Eq. and simplifying, we find

$d=\frac{p_{\text{change}}}{1-p_{\text{change}}}\frac{1}{2\pi N}\sum_{i=1}^{N} \frac{\iint p\left(x_{i}|\theta_{i}\right)p\left(y_{i}|\theta_{i}+\Delta\right)d\theta_{i}d\Delta}{\int p\left(x_{i}|\theta_{i}\right)p\left(y_{i}|\theta_{i}\right)d\theta_{i}}$,

where *p*_change_ is the observer’s prior belief that a change occurred. Finally, substituting the Von Mises noise distributions and evaluating the integrals yields [1]

$d=\frac{p_{\text{change}}}{1-p_{\text{change}}}\frac{1}{N}\sum_{i=1}^{N} \frac{I_{0}\left(\kappa_{\text{x},i}\right)I_{0}\left(\kappa_{\text{y},i}\right)}{I_{0}\left(\sqrt{\kappa_{\text{x},i}^{2}+\kappa_{\text{y},i}^{2}+2\kappa_{\text{x},i}\kappa_{\text{y},i}\cos\left(y_{i}-x_{i}\right)}\right)}$.

The observer responds “change” (*Ĉ*=1) when *d*>1 and “no change” (*Ĉ*=0) when *d*<1. This is called maximum-a-posteriori estimation and it is the strategy that maximizes accuracy if *p*_change_ equals the true frequency of change trials, i.e. 0.5 in our experiments.

This model of change detection automatically encompasses both the integration of information across items and the comparison between displays. The former is reflected by the sum in Eq. , the latter by the difference between the measurements in the first and second displays, *y_i_*−*x_i_*. Since this difference is subject to noise, errors will be made. Thus, the notion of “comparison errors” in change detection [2-5] is incorporated automatically and in a principled manner in the Bayesian model [1]. Wilken and Ma earlier proposed the decision rule max*_i_* |*y_i_*−*x_i_*|>*k*, where | ⋅ | denotes circular distance and *k* is an unknown criterion [6], but because this rule is not equivalent to Eq. , it is not optimal. Another important feature of Eq. is that the local concentration parameters in both displays, *κ*_x_*_,i_* and *κ*_y_*_,i_*, play a role. This is analogous to trial-to-trial weighting by reliability in cue integration [1,7-8]: evidence from less noisy measurements is weighted more heavily.

Model fitting and model comparison

Model predictions. In the IL model, simple expressions exist for the probability of reporting “change”: *p*(*Ĉ*=1|*C*=1)=*K*/*N*⋅(1−*ε*)+(1−*K*/*N*)⋅*g* when *N*>*K*, *p*(*Ĉ*=1|*C*=1)=1−*ε* when *N*≤*K*, and *p*(*Ĉ*=1|*C*=0)=*g*. In the other models, the probability of reporting “change” is equal to the probability that *d*>1. This probability depends on set size *N*, change magnitude Δ, and the model parameters. Because no analytical expression exists for this probability in any of the models, we estimated it for each model using Monte Carlo simulation: for every stimulus condition (specified by *N* and Δ), we drew 10,000 samples of (x,y) pairs from the generative model. For each sample, the model’s decision rule was applied, and the proportion of “change” responses among all samples was determined. This returned the model’s probability of reporting “change” on each trial for the given parameter values.

Parameter fitting. For a given model *m*, we denote the vector of model parameters by **t**. The likelihood of a specific combination of parameter values is the probability of the observer’s empirical responses given that parameter combination:$L_{m}\left(\mathbf{t}\right)=p\left(\text{data}|\mathbf{t}\right)=\prod_{k=1}^{N_{\text{trials}}} p\left(\hat{C}_{k}|N_{k},\Delta_{k},\mathbf{t}\right)$, where *N*_trials_ is the total number of trials, and *N_k_*, Δ*_k_*, and *Ĉ_k_*, denote set size, change magnitude, and the subject’s response on the *k*^th^ trial, respectively. The maximum-likelihood estimate of the parameters is the value of **t** that maximizes *L_m_*(**t**).

Bayesian model comparison. Bayesian model comparison is a method to compare models that automatically penalizes models with more free parameters [9-10]. Each model *m* produces a prediction for probability of a response *Ĉ_k_* on each trial, *p*(*Ĉ_k_*|*N_k_*,Δ*_k_*,**t**). Bayesian model comparison consists of calculating, for each model, the joint probability of a subject’s empirical responses, averaged over free parameters. This gives the model likelihood, denoted *L*(*m*):

$L\left(m\right)=p\left(\text{data}|m\right)=\int p\left(\text{data}|m,\mathbf{t}\right)p\left(\mathbf{t}|m\right)d\mathbf{t}=\int\left(\prod_{k=1}^{N_{\text{trials}}} p\left(\hat{C}_{k}|N_{k},\Delta_{k},\mathbf{t}\right)\right)p\left(\mathbf{t}|m\right)d\mathbf{t}$.

We chose the prior over the *j*^th^ parameter to be uniformly distributed on an interval of size *R_j_*. The intervals are given in Tables 1 and S1. After rewriting for numerical convenience, we arrive at $\log L\left(m\right)=L_{\max}\left(m\right)-\sum_{j=1}^{\dim\mathbf{t}} \log R_{j}+\log\int e^{\log L_{m}\left(\mathbf{t}\right)-\log L_{\max}\left(m\right)}d\mathbf{t},$where dim **t** is the number of parameters and $L_{\max}\left(m\right)=\max_{t} L_{m}\left(\mathbf{t}\right)$. We computed the integrals over parameters numerically by using the trapezoidal rule.

Supplementary results: color change detection

To examine the generality of our conclusions, we repeated the orientation change detection experiment described in the main text with color stimuli (Fig. S2). This experiment was identical except for the following differences. Stimuli were colored discs with a radius of 0.36 deg. The colors were drawn independently from 180 color values uniformly distributed along a circle of radius 60 in CIE 1976 (*L**, *a**, *b**) color space. This circle had constant luminance (*L**=58) and was centered at the point (*a**=12, *b**=13). Stimulus positions were not jittered. On each trial, set size was 1, 2, 4, or 8, with equal probabilities. Seven subjects participated (4 female, 3 male; 1 author).

The results are qualitatively consistent with those of the main experiment. We found a significant effect of set size on hit rate (*F*(3,18)=40.4, *p*<0.001) and false-alarm rate (*F*(3,18)=26.2, *p*<0.001) (Fig. S3a). The probability of reporting a change increased with change magnitude at every set size (Fig. S3b). The VP model offered the best fit to the hit and false-alarm rates (RMSE of 0.046 versus 0.059 and higher) as well as the psychometric curves (0.11 versus 0.14 and higher). The VP model also won in Bayesian model comparison (Fig. S4), surpassing the SA model by 48.4 ± 8.2 points. As for orientation, we found apparent guessing rates that increased with set size (*F*(3,18)=31.8, *p*<0.001) and were non-zero for all set sizes (*t*(6)>5.45, *p*<0.001) (Fig. S5). The VP model was better able to account for this than the next best model (the SA model), with RMSE values of 0.09 and 0.17, respectively. Parameter estimates are given in Table S1.

References

1. Keshvari S, Van den Berg R, Ma WJ (2012) Probabilistic computation in human perception under variability in encoding precision. PLoS ONE 7: e40216.

2. Awh E, Barton B, Vogel EK (2007) Visual working memory represents a fixed number of items regardless of complexity. Psych Science 18: 622-628.

3. Scott-Brown KC, Baker MR, Orbach HS (2000) Comparison blindness. Visual Cognition 7: 253-267.

4. Rensink RA (2002) Change detection. Annu Rev Psychol 53: 245-277.

5. Simons DJ, Rensink RA (2005) Change blindness: past, present, and future. Trends Cogn Sci 9: 16-20.

6. Wilken P, Ma WJ (2004) A detection theory account of change detection. J Vision 4: 1120-1135.

7. Knill DC, Pouget A (2004) The Bayesian brain: the role of uncertainty in neural coding and computation. Trends Neurosci 27: 712-719.

8. Yuille AL, Bulthoff HH (1996) Bayesian decision theory and psychophysics. In: Knill DC, Richards W, editors. Perception as Bayesian Inference. New York: Cambridge: University Press.

9. MacKay DJ (2003) Information theory, inference, and learning algorithms. Cambridge, UK: Cambridge University Press.

10. Wasserman L (2000) Bayesian model selection and model averaging. J Math Psych 44: 92-107.
